# *XPG* rs17655 G>C polymorphism associated with cancer risk: evidence from 60 studies

**DOI:** 10.18632/aging.101448

**Published:** 2018-05-20

**Authors:** Jie Zhao, Shanshan Chen, Haixia Zhou, Ting Zhang, Yang Liu, Jing He, Jinhong Zhu, Jichen Ruan

**Affiliations:** 1 Department of Hematology, The Second Affiliated Hospital and Yuying Children's Hospital of Wenzhou Medical University, Wenzhou 325027, Zhejiang, China; 2 Department of Clinical Medicine Center, The First People’s Hospital of Wenling, The Affiliated Wenling Hospital of Wenzhou Medical University, Wenling 317500, Zhejiang, China; 3 Department of Clinical Laboratory, Molecular Epidemiology Laboratory, Harbin Medical University Cancer Hospital, Harbin 150040, Heilongjiang, China; 4 Department of Pediatric Surgery, Guangzhou Institute of Pediatrics, Guangzhou Women and Children’s Medical Center, Guangzhou Medical University, Guangzhou 510623, Guangdong, China

**Keywords:** *XPG*, rs17655, polymorphism, cancer risk, meta-analysis

## Abstract

Xeroderma pigmentosum group G (XPG), a key component in nucleotide excision repair pathway, functions to cut DNA lesions during DNA repair. Genetic variations that alter DNA repair gene expression or function may decrease DNA repair ability and impair genome integrity, thereby predisposing to cancer. The association between *XPG* rs17655 G>C polymorphism and cancer risk has been investigated extensively, but the results remain contradictory. To get a more accurate conclusion, we performed a comprehensive meta-analysis of 60 case-control studies, involving 27,098 cancer cases and 30,535 healthy controls. Crude odds ratios (ORs) and 95% confidence interval (CIs) were calculated to determine the association of interest. Pooled analysis indicated that the *XPG* rs17655 G>C polymorphism increased the risk of overall cancer (CC vs. GG: OR=1.10, 95% CI=1.00-1.20; CG vs. GG: OR=1.06, 95% CI=1.02-1.11; CG+CC vs. GG: OR=1.07, 95% CI=1.02-1.12; C vs. G: OR=1.05, 95% CI=1.01-1.09). Stratification analysis by cancer type further showed that this polymorphism was associated with increased risk of gastric cancer and colorectal cancer. This meta-analysis indicated that the *XPG* gene rs17655 G>C polymorphism was associated with increased overall cancer risk, especially the risk of gastric cancer and colorectal cancer. Further validation experiments are needed to strength our conclusion.

## Introduction

Cancer-related deaths continue to rise in both developed and developing countries. In 2012, there were about 14.1 million new cancer cases and 8.2 million cancer-related deaths all over the world. Lung and breast cancer are the most common forms of cancer in human beings. Moreover, the incidences of liver, stomach and colorectal cancer are also very high in men and stomach, while cervix uteri and colorectal cancer prevail in women. Cancer is a complex disease. A variety of cancer risk factors have been recognized, such as smoking, drinking, lack of exercise, poor diet, reproductive changes, and genetic lesions [1]. Inherited genetic causations of cancer risk are mainly unidentified. Thus far, great effects have been made to discover genetic variant alleles implicated in the crucial signaling pathways, which may influence individual cancer predisposition.

Genetic DNAs of living organisms are constantly subjected to various types of damages caused by environmental agents and byproducts (e.g., reactive oxygen species) of cellular metabolic processes. To maintain genome integrity, human beings possess a number of systems for the prevention and restoration of DNA damage. Reduced DNA repair ability is a predisposing factor to cancer [2]. Five common DNA repair pathways have been identified, including nucleotide excision repair (NER), base excision repair, double-strand DNA break repair, mismatch repair, and transcription coupled repair [3,4]. Among these pathways, NER is responsible for removing damaged DNA fragments (e.g., bulky adducts) resulting from radiation or chemical agents [5,6]. In the NER pathway, at least eight vital genes [*excision repair cross-complementation group 1* (*ERCC1*), *ERCC2/ Xeroderma pigmentosum group D* (*XPD*), *ERCC3/XPB*, *ERCC4/XPF*, *ERCC5/XPG*, *XPA*, *XPC* and *XPE*/*damaged DNA-binding protein 1* (*DDB1*)] have been well studied, which participate in DNA repair, capable of preserving genetic integrity to prevent cells from malignant transformation [7].

*ERCC5/XPG* is located on chromosome 13q22-33, consisting of 15 exons and 14 introns . Its protein product is a 1,186 amino acid structure-specific endonuclease, and plays an essential role in the two incision steps of NER [4,8]. *XPG* is highly polymorphic. Among known single nucleotide polymorphisms (SNPs) in this gene, a nonsynonymous Asp1104His (rs17655, G>C) polymorphism is most frequently studied for its association with cancer risk [2,9–38]. However the results are inconsistent from study to study. Therefore, we performed this meta-analysis with all eligible publications to investigate the association between the *XPG* gene rs17655 G>C polymorphism and cancer risk.

## RESULTS

### Study characteristics

**Figure 1:**
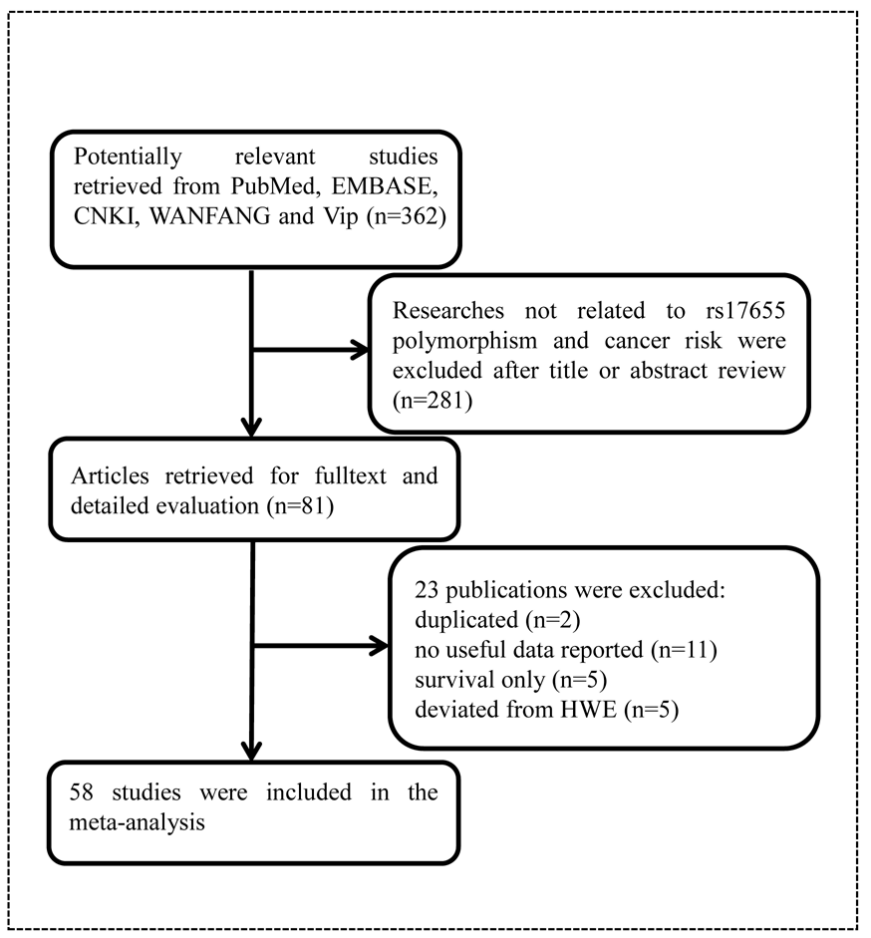
Flowchart of included publications.

As shown in Figure 1, we found 362 potentially relevant studies from PubMed, EMBASE, CNKI, WANFANG, and Vip databases. After reviewing titles and abstracts, we excluded 281 publications not investigating the association between *XPG* gene rs17655 polymorphism and cancer risk. And then, full texts of remaining articles were evaluated. Two publications [39,40] were removed for containing overlap data. We also excluded 11 publications [41–51] because no sufficient data were reported to calculate ORs and 95% CIs. Furthermore, we eliminated five publications [52–56] presenting survival data only. At last, we excluded five publications [57–61] due to deviation from HWE. In the end, 58 publications with a total of 27,098 cancer cases and 30,535 healthy controls were included in the meta-analysis. It was noteworthy that, 58 publications actually consisted of 60 case-control studies, because 2 of them included two individual studies. The characteristics of these studies were showed in Table 1. Among these publications, five focused on gastric cancer [15,22,31,37,38], 10 on breast cancer [18,29,33,34,59,62–66], four on colorectal cancer [16,20,25,67], four on lymphoma [11,21,68,69], six on bladder cancer [24,70–74], five on lung cancer [17,30,75–77], eight on skin cancer [14,23,26,32,35,78–80], three on HNC [10,81,82], two on endometrial cancer [19,83], laryngeal carcinoma [9,84], and prostate cancer [12,28]. Moreover, there was only one study for each of the following cancers: osteosarcoma [13], hepatocellular carcinoma [36], esophageal carcinoma [85], oral squamous cell carcinoma [86], sarcoma [2], cervical carcinoma [27] and brain cancer [87]. Among these case-control studies, 25 of them had quality scores higher than 9, while 35 had quality scores no more than 9. Finally, this meta-analysis contained 26 hospital-based, 31 population-based, and three mixed control studies.

**Table 1:** Characteristics of included studies in the final meta-analysis.

Name	Year	Cancer type	Region	Ethnicity	Design	Genotype	Case				Control				MAF	HWE	Score
						method	GG	CG	CC	All	GG	CG	CC	All			
Feng	2016	Gastric	China	Asian	HB	PCR-RFLP	47	85	45	177	84	107	46	237	0.42	0.260	6
Ma	2016	Breast	China	Asian	HB	PCR-RFLP	116	145	59	320	84	107	46	237	0.42	0.260	7
Du	2016	Colorectal	China	Asian	HB	TaqMan	286	459	133	878	355	405	124	884	0.37	0.623	9
Wang	2015	Breast	China	Asian	HB	PCR-RFLP	95	6	0	101	100	1	0	101	0.00	0.960	9
Bahceci	2014	B-NHL	Turkey	Others	PB	AS-PCR	59	33	1	93	43	44	9	96	0.32	0.637	4
Li	2014	Gastric	China	Asian	HB	PCR-RFLP	99	83	36	218	112	82	24	218	0.30	0.135	7
Zhu	2014	Bladder	China	Asian	HB	MassARRAY	62	160	65	287	76	139	67	282	0.48	0.825	6
Lu	2014	Larynx	China	Asian	HB	MassARRAY	53	69	54	176	78	63	36	177	0.38	0.001	8
Liu	2014	Gastric	China	Asian	HB	PCR-RFLP	99	100	39	238	120	95	23	238	0.30	0.510	8
Ruiz-Cosano	2013	BCL	Spain	Caucasian	PB	TaqMan	125	71	17	213	119	81	14	214	0.25	0.965	7
Zeng	2013	Lung	China	Asian	HB	PCR-RFLP	15	77	47	139	35	61	37	133	0.51	0.341	8
Yuan	2012	HNC	China	Asian	PB	TaqMan	108	191	95	393	234	433	217	884	0.49	0.552	12
Biason	2012	Osteosarcoma	Italy	Caucasian	HB	PCR-RFLP	75	39	16	130	141	94	15	250	0.25	0.899	8
Gil	2012	Colorectal	Poland	Caucasian	HB	PCR-RFLP	86	35	11	132	64	31	5	100	0.21	0.625	6
Berhane	2012	Prostate	India	Asian	PB	PCR-RFLP	58	72	20	150	66	75	9	150	0.31	0.039	8
Ma	2012	HNC	America	Caucasian	PB	SNPlex	648	359	52	1059	654	350	62	1066	0.22	0.099	10
Rouissi	2011	Bladder	Tunisia	African	PB	PCR	48	56	21	125	46	61	18	125	0.39	0.758	6
Ibarrola-Villava	2011	Melanoma	Spain	Caucasian	HB	TaqMan	326	222	50	598	215	140	24	379	0.25	0.85	5
Canbay	2011	Colorectal	Turkey	Others	PB	PCR-RFLP	43	34	2	79	148	83	16	247	0.23	0.352	10
Goncalves	2011	Melanoma	Brazil	Caucasian	HB	PCR-RFLP	105	77	10	192	109	74	25	208	0.30	0.031	9
Doherty	2011	Endometrial	America	Others	PB	Unknown	418	254	42	714	408	248	47	703	0.24	0.268	10
Hsu	2010	Breast	China	Asian	HB	TaqMan	76	191	134	401	129	243	159	531	0.53	0.059	8
Figl	2010	Melanoma	German, Spain	Caucasian	PB	TaqMan	703	409	74	1186	725	465	84	1274	0.25	0.420	8
Canbay	2010	Gastric	Turkey	Others	PB	PCR-RFLP	25	12	3	40	148	83	16	247	0.23	0.352	8
Li	2010	HCC	China	Asian	HB	TaqMan	174	233	93	500	151	265	91	507	0.44	0.175	11
Narter	2009	Bladder	Turkey	Others	PB	PCR-RFLP	25	28	3	56	18	19	3	40	0.31	0.505	5
Abbasi	2009	Larynx	Germany	Caucasian	PB	Real-time PCR	137	103	8	248	380	230	37	647	0.23	0.778	11
Hussain	2009	Gastric	China	Asian	PB	SNPlex	38	105	38	181	90	180	90	360	0.50	1.000	12
El-Zein	2009	HD	America	Caucasian	PB	TaqMan	104	78	16	198	127	80	12	219	0.24	0.897	10
McKean-Cowdin	2009	Brain	America	Caucasian	Mixed	TaqMan and MassARRAY	499	348	157	1004	989	657	311	1957	0.33	0.000	13
Pan	2009	Esophageal	America	Caucasian	HB	TaqMan	201	131	12	344	287	155	15	457	0.20	0.281	7
Rajaraman	2008	Breast	America	Others	PB	TaqMan	482	288	49	819	674	352	53	1079	0.21	0.423	13
Chang	2008	Lung	America	Africa American	PB	Illumina	68	119	68	255	93	138	49	280	0.42	0.858	8
Chang	2008	Lung	America	Latino	PB	Illumina	60	44	9	113	138	127	34	299	0.33	0.561	7
Pardini	2008	Colorectal	Czech	Caucasian	HB	PCR-RFLP	334	177	21	532	356	153	23	532	0.19	0.211	11
Smith	2008	Breast	America	African American	PB	MassARRAY	13	32	7	52	18	37	20	75	0.51	0.913	9
Hung	2008	Lung	World	World	Mixed	Unknown	1852	1155	209	3216	2485	1510	286	4281	0.24	0.006	10
He	2008	Cervical	China	Asian	HB	mismatch amplification PCR	71	94	35	200	67	80	53	200	0.47	0.006	8
Hooker	2008	Prostate	America	African	HB	PCR	74	119	61	254	99	142	60	301	0.44	0.484	8
Wang	2007	NMSC	Texas	Caucasian	HB	PCR	146	89	11	246	200	119	10	329	0.21	0.121	8
Povey	2007	Melanoma	Scotland	Caucasian	PB	PCR-RFLP	314	169	24	507	252	162	27	441	0.24	0.887	13
Crew	2007	Breast	America	Others	PB	Sequenom	562	371	66	999	571	409	71	1051	0.26	0.846	11
An	2007	HNC	America	Caucasian	HB	PCR	507	286	36	829	519	289	46	854	0.22	0.489	11
Jorgensen	2007	Breast	America	Others	PB	TaqMan	159	93	12	264	165	95	15	275	0.23	0.785	10
Mechanic	2006	Breast	America	African American	PB	TaqMan	231	387	139	757	231	320	123	674	0.42	0.509	9
Mechanic	2006	Breast	America	Caucasian	PB	TaqMan	771	409	69	1249	661	412	60	1133	0.23	0.685	9
Shen	2006	Breast	America	Others	PB	TaqMan	83	63	8	154	82	62	7	151	0.25	0.268	11
Sugimura	2006	OSCC	Japan	Asian	HB	PCR-RFLP	43	59	20	122	77	112	52	241	0.45	0.348	5
Garcia-Closas	2006	Bladder	Spain	Caucasian	HB	Sequencing	629	434	78	1141	607	445	84	1136	0.27	0.844	11
Li	2006	Melanoma	America	Caucasian	HB	PCR	373	206	23	602	370	206	27	603	0.22	0.805	12
Wu	2006	Bladder	America	Others	PB	TaqMan	364	225	26	615	371	211	18	600	0.21	0.064	13
Thirumaran	2006	BCC	Hungry, Romania, Slovakia	Caucasian	HB	TaqMan	325	172	32	529	330	173	30	533	0.22	0.250	11
Shen	2006	NHL	America	Others	PB	TaqMan	260	170	34	464	352	169	29	550	0.21	0.146	13
Le Morvan	2006	Sarcoma	France	Caucasian	HB	PCR-RFLP	182	107	19	308	31	21	1	53	0.22	0.227	6
Sakiyama	2005	Lung	Japan	Asian	Mixed	Pyrosequencing	300	500	202	1002	228	333	124	685	0.42	0.900	7
Shen	2005	Lung	China	Asian	PB	TaqMan	38	52	26	116	38	46	25	109	0.44	0.133	10
Weiss	2005	Endometrial	America	Caucasian	PB	PCR-RFLP	215	134	22	371	250	148	22	420	0.23	0.987	11
Blankenburg	2005	Melanoma	German	Caucasian	PB	PCR-RFLP	9	100	184	293	18	124	232	374	0.79	0.785	8
Sanyal	2004	Bladder	Sweden	Caucasian	PB	PCR-RFLP	182	109	8	299	173	91	20	284	0.23	0.102	8
Kumar	2003	Breast	Finland	Caucasian	PB	PCR-RFLP	108	96	16	220	182	107	19	308	0.24	0.540	10

MAF, minor allele frequency; HWE, Hardy-Weinberg equilibrium; B-NHL, B cell non-Hodgkin's lymphoma; BCL, B cell lymphoma; HNC, head and neck cancer; HCC, hepatocellular carcinoma; HD, Hodgkin’s disease; NMSC, non-melanoma skin cancer; OSCC, oral squamous cell carcinoma; BCC, basal cell carcinoma; HB, hospital based; PB, population based; PCR-RFLP, polymerase chain reaction-restriction fragment length polymorphism; AS-PCR, allele-specific PCR.

### Meta-analysis results

**Figure 2:**
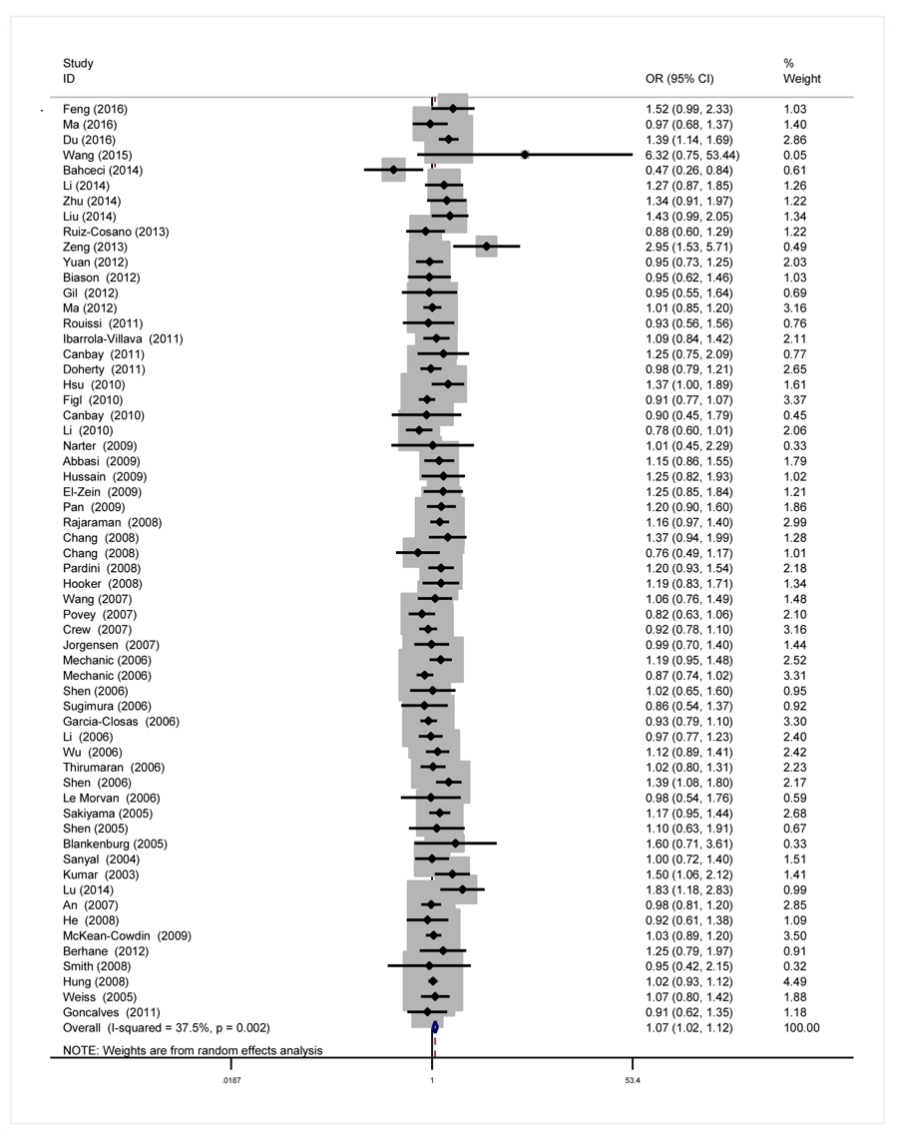
**Forest plot for the association between the *XPG* rs17655 G>C polymorphism and overall cancer risk under the dominant model (CG/CC vs. GG).** For each publication, the estimation of OR and its 95% CI was plotted with a box and a horizontal line. The diamonds represented the pooled ORs and 95% CIs.

As we can see in Table 2 and Figure 2, significant between-study heterogeneity was detected under all the genetic models in the overall analysis. Thus, we used random-effect model. After calculating crude odds ratios (ORs) and 95% confidence interval (CIs), we found that *XPG* gene rs17655 G>C polymorphism was associated with increased overall cancer susceptibility (CC vs. GG: OR=1.10, 95% CI=1.00-1.20, *P*=0.032; CG vs. GG: OR=1.06, 95% CI=1.02-1.11, *P*=0.013; CG+CC vs. GG: OR=1.07, 95% CI=1.02-1.12, *P*=0.004; C vs. G: OR=1.05, 95% CI=1.01-1.09, *P*=0.011). Stratification analysis further indicated that the *XPG* gene rs17655 G>C polymorphism was associated with increased risk of gastric cancer (CC vs. GG: OR=1.53, 95% CI=1.16-2.01, *P*=0.002; CG vs. GG: OR=1.25, 95% CI=1.02-1.53, *P*=0.030; CG+CC vs. GG: OR=1.32, 95% CI=1.09-1.60, *P*=0.005; C vs. G: OR=1.23, 95% CI=1.06-1.42, *P*=0.005) and colorectal cancer (CG vs. GG: OR=1.30, 95% CI=1.12-1.51, *P*=0.001; CG+CC vs. GG: OR=1.28, 95% CI=1.11-1.48, *P*=0.001; C vs. G: OR=1.16, 95% CI=1.05-1.30, *P*=0.011) (Supplemental Figure 1). We also checked the association in Asian (18 studies) and Caucasian (24 studies), among which ethnic groups studies were enriched. Interestingly, we only observed significant association in Asian (CC vs. GG: OR=1.25, 95% CI=1.05-1.49, *P*=0.013; CG vs. GG: OR=1.20, 95% CI=1.06-1.35, *P*=0.002; CG+CC vs. GG: OR=1.21, 95% CI=1.07-1.38, *P*=0.005; C vs. G: OR=1.13, 95% CI=1.03-1.23, *P*=0.005). Moreover, the association remained significant in the subgroups with quality score ≤ 9 (CC vs. GG: OR=1.20, 95% CI=1.04-1.39, *P*=0.015; CG vs. GG: OR=1.09, 95% CI=1.00-1.18, *P*=0.033; CG+CC vs. GG: OR=1.11, 95% CI=1.02-1.21, *P*=0.018; C vs. G: OR=1.07, 95% CI=1.01-1.15, *P*=0.065) and hospital-based studies (CC vs. GG: OR=1.19, 95% CI=1.02-1.39, *P*=0.028; CG vs. GG: OR=1.10, 95% CI=1.01-1.20, *P*=0.032; CG+CC vs. GG: OR=1.12, 95% CI=1.02-1.22, *P*=0.009; C vs. G: OR=1.09, 95% CI=1.02-1.16, *P*=0.007).

**Table 2:** Meta-analysis of the association between *XPG* gene rs17655 G>C polymorphism and overall cancer risk.

Variables	No. of	Homozygous			Heterozygous			Recessive			Dominant			Allele	
	studies	CC vs. GG			CG vs. GG			CC vs. CG+GG			CG+CC vs. GG			C vs. G	
		OR (95% CI)	*P*^het^		OR (95% CI)	*P*^het^		OR (95% CI)	*P*^het^		OR (95% CI)	*P*^het^		OR (95% CI)	*P*^het^
All	60	**1.10(1.00-1.20)**	0.001		**1.06(1.02-1.11)**	0.040		1.04(0.97-1.12)	0.028		**1.07(1.02-1.12)**	0.002		**1.05(1.01-1.09)**	0.000
Cancer type															
Gastric	5	**1.53(1.16-2.01)**	0.407		**1.25(1.02-1.53)**	0.793		1.30(0.93-1.82)	0.131		**1.32(1.09-1.60)**	0.755		**1.23(1.06-1.42)**	0.288
Breast	11	1.10(0.95-1.27)	0.613		1.08(0.95-1.22)	0.047		1.04(0.92-1.19)	0.768		1.08(0.95-1.22)	0.036		1.04(0.96-1.14)	0.073
Colorectal	4	1.24(0.96-1.59)	0.395		**1.30(1.12-1.51)**	0.395		1.06(0.84-1.34)	0.401		**1.28(1.11-1.48)**	0.554		**1.16(1.05-1.30)**	0.875
Lymphoma	4	1.13(0.57-2.24)	0.049		0.98(0.69-1.41)	0.022		1.17(0.66-2.08)	0.110		0.97(0.65-1.46)	0.004		0.98(0.69-1.39)	0.001
Bladder	6	0.97(0.71-1.33)	0.177		1.03(0.92-1.16)	0.520		0.93(0.70-1.24)	0.193		1.02(0.91-1.14)	0.588		1.00(0.91-1.09)	0.636
Lung	6	1.26(0.92-1.73)	0.007		1.13(0.93-1.37)	0.051		1.12(0.92-1.37)	0.136		1.16(0.94-1.43)	0.011		1.11(0.96-1.28)	0.012
HNC	3	0.88(0.71-1.09)	0.819		1.01(0.90-1.14)	0.898		0.90(0.74-1.10)	0.684		0.99(0.88-1.11)	0.944		0.97(0.89-1.06)	0.984
Others	13	1.09(0.88-1.36)	0.014		1.04(0.95-1.14)	0.411		1.07(0.87-1.31)	0.014		1.05(0.95-1.15)	0.226		1.05(0.96-1.15)	0.051
Skin	8	0.96(0.75-1.23)	0.175		0.97(0.88-1.06)	0.793		0.96(0.79-1.17)	0.254		0.96(0.88-1.05)	0.657		0.97(0.90-1.04)	0.427
Ethnicity															
Asian	18	**1.25(1.05-1.49)**	0.003		**1.20(1.06-1.35)**	0.031		1.10(0.97-1.25)	0.044		**1.21(1.07-1.38)**	0.005		**1.13(1.03-1.23)**	0.002
Caucasian	24	0.98(0.87-1.10)	0.254		1.01(0.95-1.06)	0.437		0.97(0.86-1.09)	0.230		1.00(0.95-1.05)	0.575		0.99(0.95-1.04)	0.590
Quality score															
>9	25	0.98(0.90-1.07)	0.872		1.04(0.99-1.09)	0.341		0.97(0.90-1.05)	0.932		1.03(0.98-1.08)	0.267		1.01(0.98-1.05)	0.447
≤9	35	**1.20(1.04-1.39)**	0.000		**1.09(1.00-1.18)**	0.023		1.10(0.98-1.24)	0.002		**1.11(1.02-1.21)**	0.001		**1.07(1.01-1.15)**	0.000
Design															
HB	26	**1.19(1.02-1.39)**	0.002		**1.10(1.01-1.20)**	0.031		1.09(0.97-1.24)	0.034		**1.12(1.02-1.22)**	0.004		**1.09(1.02-1.16)**	0.003
PB	31	1.03(0.91-1.17)	0.079		1.04(0.97-1.10)	0.185		1.00(0.90-1.12)	0.118		1.03(0.97-1.10)	0.069		1.02(0.97-1.07)	0.022
Mixed	3	1.04(0.91-1.18)	0.376		1.05(0.97-1.13)	0.690		1.01(0.90-1.14)	0.550		1.04(0.97-1.12)	0.504		1.03(0.97-1.09)	0.431

HNC, Head and Neck cancer; OR, odds ratio; CI, confidence interval; Het, heterogeneity.

### Publication Bias

**Figure 3:**
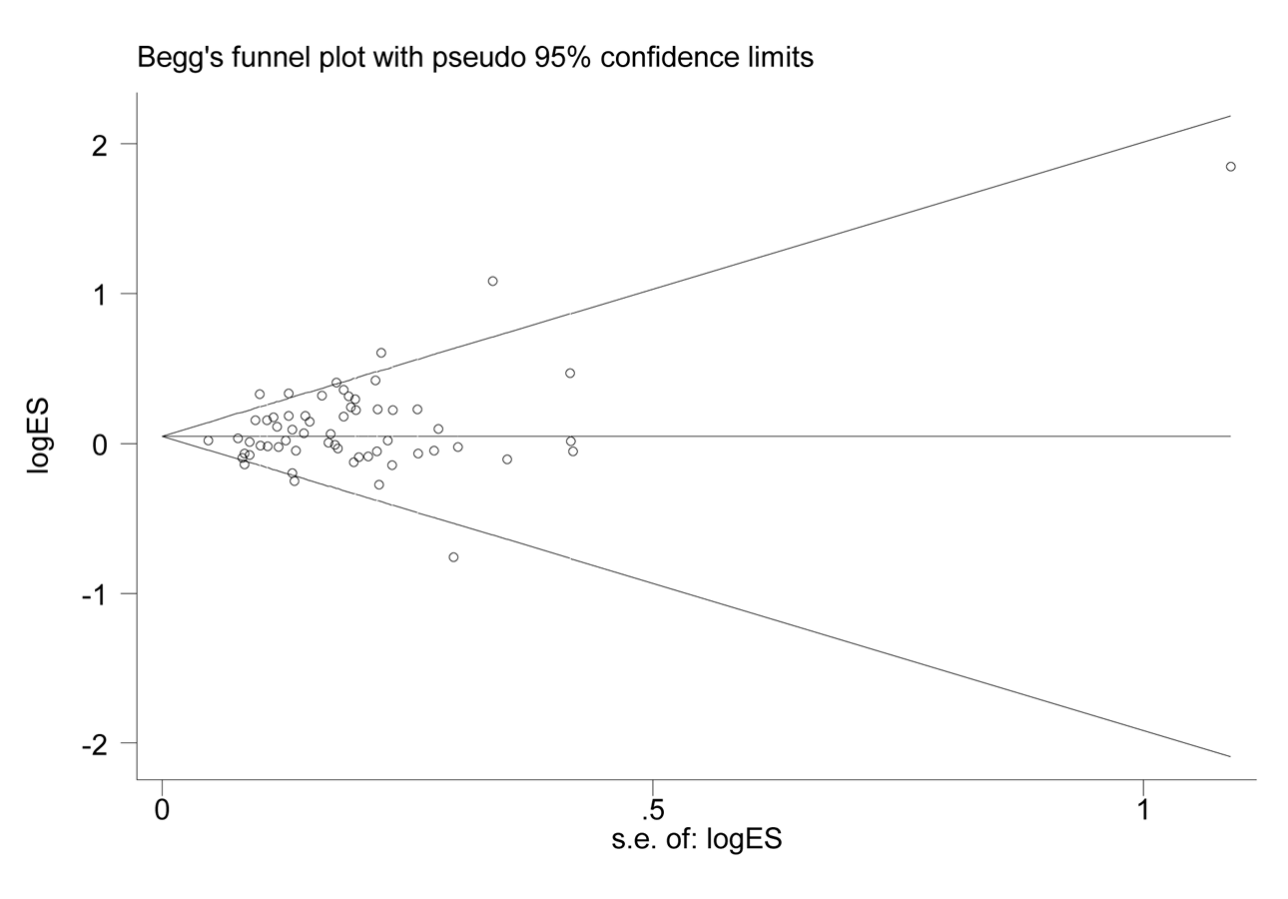
Funnel plot for the association between *XPG* gene rs17655 G>C polymorphism and overall cancer risk under the dominant model (CG/CC vs. GG).

Symmetry in the funnel plot (Figure 3) suggested that there was no significant publication bias in this meta-analysis (CC vs. GG: *P*=0.808; CG vs. GG: *P*=0.050; CC vs. CG+GG: *P*=0.806; CG+CC vs. GG: *P*=0.047; C vs. G: *P*=0.240).

## DISCUSSION

In the current meta-analysis, we estimated the association between the *XPG* gene rs17655 G>C polymorphism and cancer risk based on 60 eligible case-control studies with a total of 27,098 cancer cases and 30,535 healthy controls. Pooled risk estimates revealed that this polymorphism was significantly associated with an increased risk of overall cancer, especially with the risk of gastric cancer and colorectal cancer.

The etiology of cancer is multifactorial [1]. Abnormal accumulation of DNA mutations caused by a variety of factors might eventually trigger carcinogenic process [68]. Thus, properly repairing DNA damages in time to ensure genome stability and integrity is essential to prevent cancer. NER system includes two pathways: global genome repair and transcription-coupled repair, in both of which XPG plays a crucial role [6–8]. *XPG* gene, one of the eight vital genes in the NER pathway, is responsible for recognizing and excising DNA lesions on the 3’ side [3,4]. Loads of SNPs have been identified in the *XPG* gene over the past decades, among which the rs17655 polymorphism has revoked great attention for its association with cancer risk. The rs17655 polymorphism, leading to the replacement of aspartate with histidine at codon 1104 in ERCC5 protein, may cause an alteration in the protein function, thereby likely affecting DNA repair ability, genome integrity, and cancer predisposition.

Numerous studies were performed to explore the association between the rs17655 polymorphism and the risk of various types of cancer. Feng et al. [22] carried out a study in 2016 to investigate the roles of three SNPs (rs2094258, rs751402 and ra17655) in the *XPG* gene, consisting of 177 patients and 237 controls. They found that the rs17655 polymorphism was associated with an increased risk of gastric cancer. This association was reconfirmed in different types of cancer, including breast cancer by Hsu et al. [29] with 401 cases and 531controls, colorectal carcinoma by Du et al. [20] with 878 cases and 884 controls, lung cancer by Chang et al. [17] with 255 cases and 280 controls, as well as cancer of other types. However, opposite results were also frequently reported. A population-based case-control study containing 196 gastric cases and 397 controls subjects conducted by Hussain et al. [31] revealed that the *XPG* rs17655 polymorphism might be associated with reduced gastric cancer risk. Additionally, Ruiz-Cosano et al. [68] reported that this polymorphism did not seem to play a major role in lymphoma susceptibility after studying 213 cases and 214 controls. Ma et al. [62] selected 320 cases and 294 controls and found that the rs17655 polymorphism might not confer susceptibility to breast cancer after adjusting for potential confounding factors. Several meta-analyses were also conducted, and unfortunately the results were still inconsistent [88–91]. As contradictory results were produced, we performed this meta-analysis to draw a more precise conclusion by including larger sample size and different cancer types from 60 studies. Our result indicated that this polymorphism may increase the risk of overall cancer, especially the risk of gastric cancer and colorectal cancer. The biological function of the rs17655 remains obscure. This polymorphism has been intensively studied for its association with cancer risk as a tagger. It was predicated to be a harmful variant by a sequence homology-based tool [92]. Moreover, its functional potential was further confirmed by SIFT algorithms (scale invariant feature transform) and SNPs3D tools (http://compbio.cs.queensu.ca/F-SNP/) [93]; however, solid *in vitro* and *in vivo* data are needed to elucidate biological function of this variant.

There are advantages that strengthened the robustness of our findings. First, we searched five databases to include most of the publications written in English or Chinese. The large sample size provided adequate statistical power. Second, stratified analyses were performed by cancer type, quality score, and source of control. Third, we used the Begg’s funnel plot and Egger’s linear regression test to assess the possible publication bias.

However, several limitations still existed in this meta-analysis. Firstly, selection bias might occur because only publications written in English or Chinese were included. Researches in other languages were missed. Secondly, the number of individual studies for some cancer types, like HNC and prostate cancer (<5 studies), may be inadequate. Third, more than half of included studies had relative low quality scores (≤ 9). Our results should be interpreted cautiously. Further studies with high quality scores are needed to verify the real association.

Additionally, age, sex, living habits, virus infections or some environmental factors may also influence cancer risk. Our findings based on unadjusted estimates for lack of access to original data might suffer from potential confounding bias. Therefore, the results should be interpreted with caution. Finally, lack of biological evidence of the implication of the rs17655 polymorphism in cancer is also a drawback of the study. Mechanistic studies of the rs17655 polymorphism with cancer should be performed in the future.

In conclusion, this meta-analysis suggests that the *XPG* rs17655 G>C polymorphism is significantly associated with an increased overall cancer risk, especially with the risk of gastric cancer and colorectal cancer. Moreover, large-scale, well-designed studies in different cancers should be conducted to corroborate our findings.

## MATERIALS AND METHODS

### Publication search

We searched for relevant articles using the following terms: “*ERCC5* or *XPG*”, “polymorphism or variant”, and “cancer or carcinoma or neoplasm or malignance” in PubMed, EMBASE, CNKI, WANFANG, and Vip databases (the last search was performed on June 17, 2016). We also manually searched the references of the retrieved publications for additional relevant eligible studies.

### Inclusion and Exclusion criteria

The publications contained in the meta-analysis had to meet the following criteria: (1) the study was only written in English or Chinese; (2) the study investigated the association between the *XPG* gene rs17655 polymorphism and the risk of one or more types of cancer; (3) case-control study. If studies had overlapping subjects, the publication including the largest number of individuals were selected.

Exclusion criteria were as follows (1) the study did not report sufficient genotype data to calculate odds ratio (OR) and 95% confidence interval (CI); (2) the study included survival data only. (3) the genotype frequencies of the rs17655 G>C and other polymorphisms were deviated from Hardy-Weinberg equilibrium (HWE) in the controls.

### Data Extraction and quality assessment

Two investigators (Chen SS and Zhao J) extracted the following information from each publication independently: first author, publication year, cancer type, country of origin, race, genotyping method, source of controls (hospital-based, population-based and mixed), the genotype counts of cases and controls for the rs17655 G>C polymorphism. We also calculated the score of each publication based on the quality score assessment as described before [94]. All contradictory information was discussed when necessary.

### Statistical analysis

We evaluated crude ORs and 95% CIs to assess the association between *XPG* rs17655 G>C polymorphism and overall cancer risk under the homozygous (CC vs. GG), heterozygous (CG vs. GG), recessive (CC vs. CG+GG), dominant (CG+CC vs. GG), and allele contrast (C vs. G) models. We carried out stratification analyses by cancer type (if one cancer type were investigated in less than three studies, we termed this type as “others”), score (>9 and ≤9), and study design (if a study contained both hospital-based controls and population-based subjects, we termed the study design as “mixed”). We also calculated between-study heterogeneity using the Chi square-based Q-test. When *P*>0.1 indicating lack of heterogeneity, a fixed-effect model was adopted. Otherwise, a random-effect model would be applied [94]. The potential publication bias was evaluated by Begg’s funnel plot [95] and Egger’s linear regression test [96]. All of the *P* values were two-tailed. *P*<0.05 was considered statistically significant. All data analyses were performed by the STATA software (Version 12.0; Stata Corporation, College Station, TX).

## Supplementary Material

Supplementary File
