# Supplementary material for: XPG rs17655 G>C polymorphism associated with cancer risk: evidence from 60 studies
Source: Aging (Albany NY). 2018 May 20;10(5):1073–88. doi: 10.18632/aging.101448 (PMC5990387; doi:10.18632/aging.101448)
Supplement: Supplementary File [file aging-10-101448-s001.pdf]

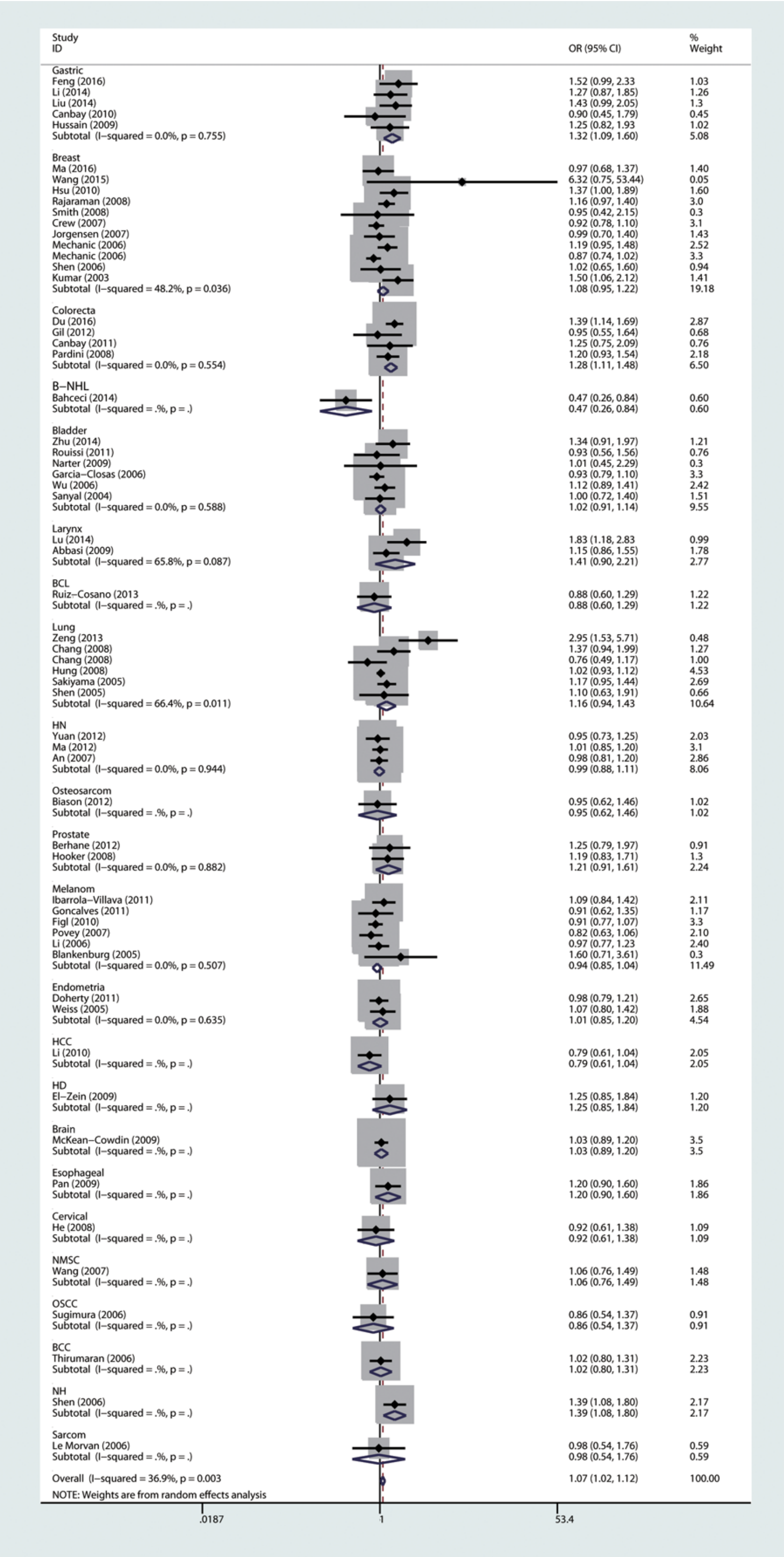

Supplemental Figure 1. Stratification analysis for the association between the *XPG* rs17655 G>C polymorphism and cancer risk by cancer type under the dominant model. For each publication, the estimation of OR and its 95%CI was plotted with a box and a horizontal line. The diamonds represented the pooled ORs and 95% CIs. Note: P value could not be calculated for single study.
